# Health impacts of takeaway management zones around schools in six different local authorities across England: a public health modelling study using PRIMEtime

**DOI:** 10.1186/s12916-024-03739-8

**Published:** 2024-11-19

**Authors:** Nina Trivedy Rogers, Ben Amies-Cull, Jean Adams, Michael Chang, Steven Cummins, Daniel Derbyshire, Suzan Hassan, Matthew Keeble, Bochu Liu, Antonieta Medina-Lara, Bea Savory, John Rahilly, Richard Smith, Claire Thompson, Martin White, Oliver Mytton, Thomas Burgoine

**Affiliations:** 1 MRC Epidemiology Unit, University of Cambridge School of Clinical Medicine, Box 285 Institute of Metabolic Science, Cambridge Biomedical Campus, Cambridge, CB2 0QQ UK; 2 Population Health Innovation Lab, Department of Public Health, Environments & Society, London School of Hygiene & Tropical Medicine,, London, WC1H 9SH UK; 3 Nuffield Department of Primary Care Health Sciences, University of Oxford, Oxford, UK; 4 Office for Health Improvement and Disparities, Department of Health and Social Care, London, UK; 5 Department of Public Health and Sport Sciences, Faculty of Health and Life Sciences, University of Exeter, Exeter, UK; 6 Department of Marketing, Faculty of Business and Economics, University of Antwerp, Antwerp, Belgium; 7 Key Laboratory of Ecology and Energy-Saving Study of Dense Habitat (Ministry of Education of China), Tongji University, Shanghai, China; 8 Department of Urban Planning, College of Architecture and Urban Planning, Tongji University, Shanghai, China; 9 UCL Great Ormond Street Institute of Child Health, 30 Guilford Street, London, WC1N 1EH UK; 10 School of Health and Social Work, University of Hertfordshire, Hertfordshire, UK

**Keywords:** Takeaway (‘fast-’) food outlets, Management zones around schools, Health impact modelling, Body mass index, Obesity, Non-communicable diseases, Quality-adjusted life years, Healthcare cost savings, PRIMEtime

## Abstract

**Background:**

In England, the number of takeaway food outlets (‘takeaways’) has been increasing for over two decades. Takeaway management zones around schools are an effective way to restrict the growth of new takeaways but their impacts on population health have not been estimated.

**Methods:**

To model the impact of takeaway management zones on health, we used estimates of change in and exposure to takeaways (across home, work, and commuting buffers) based on a previous evaluation suggesting that 50% of new outlets were prevented from opening because of management zones. Based on previous cross-sectional findings, we estimated changes in body mass index (BMI) from changes in takeaway exposure, from 2018 to 2040. We used PRIMEtime, a proportional multistate lifetable model, and BMI change to estimate the impact of the intervention, in a closed-cohort of adults (25–64 years), on incidence of 12 non-communicable diseases, obesity prevalence, quality-adjusted life years (QALYs), and healthcare costs saved by 2040 in six local authorities (LAs) across the rural–urban spectrum in England (Wandsworth, Manchester, Blackburn with Darwen, Sheffield, North Somerset, and Fenland).

**Results:**

By 2031, compared to no intervention, reductions in outlet exposure ranged from 3 outlets/person in Fenland to 28 outlets/person in Manchester. This corresponded to mean per person reductions in BMI of 0.08 and 0.68 kg/m^2^, respectively. Relative to no intervention, obesity prevalence was estimated to be reduced in both sexes in all LAs, including by 2.3 percentage points (PP) (95% uncertainty interval:2.9PP, 1.7PP) to 1.5PP (95%UI:1.9PP, 1.1PP) in males living in Manchester and Wandsworth by 2040, respectively. Model estimates showed reductions in incidence of disease, including type II diabetes (e.g. 964 (95% UI: 1565, 870) fewer cases/100,000 population for males in Manchester)), cardiovascular diseases, asthma, certain cancers, and low back pain. Savings in healthcare costs (millions) ranged from £1.65 (95% UI: £1.17, £2.25)/100,000 population in North Somerset to £2.02 (95% UI: £1.39, £2.83)/100,000 population in Wandsworth. Gains in QALYs/100,000 person were broadly similar across LAs.

**Conclusions:**

Takeaway management zones in England have the potential to meaningfully contribute towards reducing obesity prevalence and associated healthcare burden in the adult population, at the local level and across the rural–urban spectrum.

**Supplementary Information:**

The online version contains supplementary material available at 10.1186/s12916-024-03739-8.

## Background

Meals purchased out-of-home, including foods from takeaway food outlets (‘takeaways’), are typically energy dense and high in sugar and salt, but low in micronutrients, and tend to be served in large portions [1–3]. Consumption of takeaway food is associated with lower diet quality, higher energy intake and body mass index (BMI), weight gain, and greater risk of obesity [4, 5]. This may be a result of passive over-consumption of takeaway foods, which bypass regular human satiety mechanisms [6]. In turn, poor diet and excess weight are risk factors for diseases including type II diabetes and cardiovascular disease [7–9].

Neighbourhood food environments have become a focus for public health action as they may encourage unhealthy dietary behaviours [10]. Residential exposure to takeaways has been associated with takeaway food consumption, BMI, and risk of obesity in adults living in England and elsewhere [11–15] and also in children [16]. However, this relationship has not been observed in all studies [17]. Most evidence linking takeaway exposure to BMI has emerged from observational, cross-sectional studies [18]. Longitudinal studies have been conducted outside the UK but have been fewer in number and their inconsistent findings suggest differences between countries in this relationship [15, 19]. Differences between takeaway exposure and change in BMI have been hypothesised to differ between neighbourhoods in urban and rural areas due to differences in the structure of the built environment and in the dietary patterns and levels of overweight of residents [20, 21]. However, one study in the Netherlands found that takeaway exposure within 1 km of the home was associated with higher BMI in both rural and urban populations [22].

In the UK, takeaways continue to increase in number, with 47,961 registered in 2023, equating to a 2.8% increase per year from 2018 onwards [23]. In one study in Norfolk, England, longer term data suggests that the density of takeaways increased by approximately 43% over an 18-year period from 1990 to 2008 [24]. Furthermore, an increase in nominal expenditure on takeaway food from £7.9 billion in 2009 to £9.9 billion in 2016 has previously been reported by the takeaway food industry [24].

Takeaways have been shown to cluster within walking distance of schools in England and other countries [25, 26]. In England, and often with the stated intention of improving health, urban planners can use existing powers to prevent new takeaways opening, thereby limiting growth in people’s future exposure to takeaways. These ‘takeaway management zones’ are commonly centred on schools, for example where no new takeaways are permitted within 400 m radius of a school site. These are also sometimes referred to by local authorities (LAs) as takeaway ‘exclusion zones’. It has previously been estimated that management zones in England covered an average of 17% of land area in the LAs in which they have been adopted, a significant spatial footprint with capacity therefore to affect whole populations, in addition to children [27]. A recent study showed that implementation of management zones was associated with a 54% reduction in the number of new takeaways at up to six years post-intervention [28]. This is likely due to a combination of a decrease in the number of planning applications submitted for new takeaways, and an increase in the percentage of these applications being rejected, which was also observed in these areas [27]. However, the extent to which takeaway management zones around schools may benefit population health due to this retail change has not been explored.

Evaluating the future health impacts of takeaway management zones around schools is important to inform uptake and implementation. A lack of evidence in this regard has been cited as a barrier to adoption [29, 30], and future studies should be designed as best as possible to establish causal relationships. Such evidence is also important in the defence of takeaway management zones against legal challenges as the proportionality principle requires potential harms to private interests be offset by the likelihood of benefits to the public [31]. However, as the future is uncertain, this is inherently difficult. The potential positive health impacts of takeaway management zones may also accrue over a long time-period, making it challenging and untimely to *observe* the effects of policy adoption. However, mathematical modelling can be used to predict future impacts and help inform decision making [32]. The PRIMEtime model is a multistate lifetable that has been used to estimate the health impacts of other interventions such as the UK soft drink industry levy and restrictions of television advertising of unhealthy foods to children [33]. In this study, we aimed to estimate the future health impacts in the adult population, to 2040, of the adoption of takeaway management zones around schools in six different LAs across England. Changes in levels of exposure to takeaway outlets and population BMI were also estimated.

## Methods

### Takeaway outlet data

Here, we define takeaways as food outlets which sell hot food intended to be consumed off the premises. They are also known as use class A5 (otherwise known as class *Sui Generis* since 2020) within the urban planning system in England [34].

### Scenarios of restricted future takeaway growth

We used data from a previously published forecast model to the year 2031 of mean changes in population exposure to takeaways in absence of the intervention, i.e. under business-as-usual conditions [35]. Briefly, the model used historical observed rates of growth in takeaways in non-adopter LAs that were similar in terms of urban–rural class to six purposively selected LAs: Wandsworth, Manchester, Sheffield, Blackburn with Darwen, North Somerset, and Fenland (Table 1). These LAs were selected to represent classes across the rural–urban spectrum and to ensure geographical breadth across England. The selection also represented LAs that were either adopters of management zones around a similar year (Wandsworth, Manchester, and Blackburn with Darwen) or hypothetical adopters (Sheffield, North Somerset, and Fenland). Consequently, we also focus on these six LAs in our analysis. Population exposure to takeaways within LAs was measured across home, work, and commuting domains, using census travel to work data [35]. Exposures across these three domains were summed unless individuals worked and lived within the same output area or they reported working from home, in which case the exposure within the home buffer was counted twice. Exposure to takeaways has previously been measured across these same three domains [11]. Because the association between takeaway exposure and BMI has not been estimated in all age groups, and as it may differ between younger and older adults, we restricted our study to adults in their early to midlife (25–64 years old) at baseline, corresponding to the age group studied in previous UK work relating takeaway exposure to BMI [11].

**Table 1:** Demographic and urban–rural characteristics of six specified local authorities

Local authority	Rural–Urban Classification	Income deprivation quintile^a^	Population-weighted mean BMI (kg/m^2^)^b^		Population density^c^	Gender of adult population aged 25–64 years (*N*)^d^			Age group *N* (%)	
			Males	Females		Males	Female	All	25–44 years	45–64 years
Wandsworth	Urban with major conurbation (London)	3	29.4	28.5	9560	99,161	106,709	205,870	142,344 (69.1)	63,526 (30.9)
Manchester	Urban with major conurbation (non-London)	1	28.8	28.6	4773	149,682	139,056	288,738	187, 934 (65.1)	100,804 (34.9)
Sheffield	Urban with minor conurbation	2	29.6	28.5	1513	145,487	145,249	290,736	157, 524 (54.2)	133,212 (45.8)
Blackburn with Darwen	Urban with city and town	1	30.1	29.4	1129	38,110	37,792	75,902	38,110 (50.2)	37,792 (49.8)
North Somerset	Urban with significant rural	3	27.9	27.5	580	133,990	140,745	274,735	117,521 (42.8)	157,214 (57.2)
Fenland	Largely or mainly rural	2	26.7	25.8	188	25,282	25,583	50,865	23,405 (46.0)	27,460 (54.0)

^a^Income deprivation quintile is for the whole population. Most deprived quintile = 1

^b^Mean BMI is calculated as the weighted average BMI by age and sex across each local authority population in 2015 (see: https://www.ons.gov.uk/peoplepopulationandcommunity/personalandhouseholdfinances/incomeandwealth/datasets/mappingincomedeprivationatalocalauthoritylevel)

^c^Population/square km (in relation to the whole population) https://www.ons.gov.uk/datasets/TS006/editions/2021/versions/4?f=get-data

^d^Numbers are based on PRIMEtime data in 2015

In this study, relative to business-as-usual growth, we modelled impacts of policy adoption under a realistic scenario where there was a 50% reduction in new takeaways, informed by previous research [28]. While takeaway management zones were adopted between 2015 and 2017, we aligned implementation dates to 2018 to allow for comparison between LAs. We also carried out sensitivity analyses under perfect and optimistic implementation scenarios, whereby there was a 100% and 75% reduction in new takeaways following the intervention, respectively. We assumed the policy was in place between 2018 and 2031, but given that forecasting in the longer term may lead to less precise estimates, we assumed that any differences between business as usual and the intervention remained constant thereafter to 2040. Estimation of lower and upper confidence intervals was performed in R version 4.1.0.

### Relationship between change in takeaway exposure and BMI

In a previous study of UK adults aged 29–62 years, those most exposed (quartile 4) to takeaways across home, work, and commuting domains had on average 1.21 kg/m^2^ (95% CI 0.68, 1.74) greater BMI than those least exposed (quartile 1) [11]. This is equivalent to an increase in BMI of 0.0241 kg/m^2^ for each additional takeaway a person is exposed to on a regular basis (unpublished results). This magnitude of association was similar to findings from a separate study using data from the Fenland Study, which showed 0.14 kg/m^2^ higher BMI per five additional takeaways exposed to [36]. We used this figure to estimate mean change in BMI attributable to change in per person exposure to takeaways within each LA for adults aged 25–64 years. Differences between takeaway exposure and change in BMI have been hypothesised to differ between neighbourhoods in urban and rural areas due to differences in the structure of the built environment and in the dietary patterns and levels of overweight of residents [20, 21]. However, one study in the Netherlands found that takeaway exposure within 1 km of the home was associated with higher BMI in both rural and urban populations [22].

### Health impact modelling using PRIMEtime

We used PRIMEtime, a proportional multistate lifetable model, to simulate the impact of observed changes in BMI on a range of diet-related chronic diseases and other health outcomes. The PRIMEtime model works by simulating a change in obesity prevalence attributable to the intervention. It then estimates changes in incidence of specified BMI-related diseases and in disease-specific death rates while keeping deaths unrelated to obesity stable. The business-as-usual future BMI distribution was based on data from the year 2018. The relative risk associating BMI to each condition was defined per unit of BMI. In our main analysis, we estimated the health impacts for a closed cohort of adults aged 25–64 years across 22 years (2018–2040) for each of the six LAs, assuming realistic implementation. We used Microsoft Excel to conduct 1000 runs of a Monte Carlo analysis in PRIMEtime, to estimate lower and upper uncertainty intervals (UI) of cases for 12 BMI-related non-communicable diseases and their associated quality adjusted life years (QALYs) benefits and healthcare cost saving outcomes.

Diseases related to BMI that were modelled in PRIMEtime were type II diabetes, ischaemic heart disease (IHD), atrial fibrillation/flutter, stroke, hypertensive heart disease, asthma, colon and rectum cancer, oesophageal cancer, breast cancer (females only), osteoarthritis (hip and knee), and low back pain. For estimating healthcare costs in PRIMEtime, disease-specific costs for each modelled disease are based on a range of routine national datasets including hospital episodes statistics admissions data; furthermore, a detailed description of the model, including how healthcare costs are attributed to disease burden, has been published previously [37]. QALYs were also estimated using utility weights and discounted using published National Institute for Health and Care Excellence (NICE) rates at a flat 3.5% for all health outcomes and costs [38]. Future disease costs not related to the intervention were not included in any estimates of cost savings. In our results section, we show total healthcare cost savings and QALYs gained in specific LAs, and we also adjust the values by dividing them by the number of adults aged 25–64 living in a specific LA in 2018 and then multiplying by 100,000 to show values per 100,000 population.

**Fig. 1:**
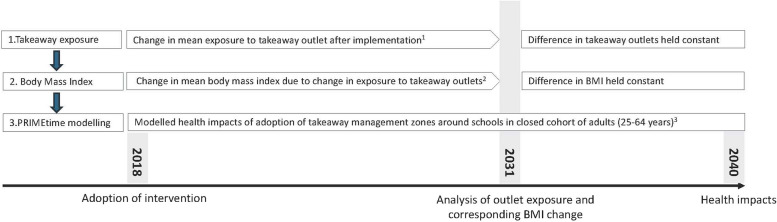
Strategic diagram of analysis strategy. The symbol “^1^” indicates the following: change in mean exposure to takeaways (by 2031) is calculated by comparing the difference in outlet exposure from a business-as-usual model (see Liu et al. 2024) to an intervention that reduces outlet growth between by 50%. The symbol “^2^” indicates the following: for each additional takeaway an individual is exposed to, mean BMI increases by 0.0241 kg/m^2^ (see Burgoine et al. 2024). The symbol “^3^” indicates the following: For PRIMEtime modelling, the oldest age of a cohort member would be aged 64 years old at baseline (2018) and would be 86 years old by 2040. Some adults will be lost to follow-up, for example due to premature mortality

Our modelling assumed that during the course of the study, the BMI of adults aged 65 years and older was no longer influenced by a change in exposure to takeaways, with any differences in BMI between business-as-usual and the intervention scenario remaining constant in this cohort after this point. This decision was informed by recent literature on dietary intake that showed how in the UK younger adults (aged 19–29 years) were five times as likely to eat takeaway meals at home relative to adults aged over 70 years [39]. Thus, we took a cautious approach to ensure we did not overestimate any potential impact of reduction in exposure to takeaway outlets on BMI. Disease incidence estimates were based on time lags from the effect of BMI changes from takeaway management zones. A time lag of five years was assigned for diabetes and cardiovascular diseases based on World Health Organization estimates of reversal of stroke and heart disease [40], 10 years for cancer based on cohort study findings examining intentional weight loss and breast cancer risk [41], and one year for all other diseases. A schematic diagram of our analytical strategy is shown in Fig. 1.

In a sensitivity analysis, relative to business-as-usual growth, we modelled impacts of policy adoption under a ‘perfect implementation’ scenario where there was a total (100%) reduction in new takeaway growth in the takeaway management zones.

## Results

Demographic characteristics of the six LAs are described in Table 1. Wandsworth, a major urban LA in London, has a population density approximately 50 times higher than Fenland, an LA that is mainly rural. Urban LAs had populations with a higher proportion of younger adults (aged 25–44 years). For example, 69% of the population of Wandsworth is within this age group, whereas it constitutes only 46% of Fenland’s population.

### Takeaway exposure following adoption of management zones

Mean exposure to takeaways at baseline varied between the six LAs, with populations of rural LAs (North Somerset and Fenland) exposed on average to approximately two thirds fewer takeaways than in other more urban LAs (Table 2). Adoption of takeaway management zones, assuming realistic implementation, led to exposure to fewer takeaways on average, per person, across all LAs relative to business as usual, with the highest absolute reductions in more urban areas. For example, in Manchester, realistic implementation was estimated to reduce average exposure to 28.4 (95% CI 25.8, 31.0) fewer new takeaways per person by 2031, relative to business as usual. Reductions in takeaway exposure were lower in other LAs, with exposure to 3.2 (95% CI 1.98, 4.43) fewer new takeaways in Fenland, relative to business as usual. Reductions were stronger under an optimistic implementation scenario, and strongest under perfect implementation, where we estimated in Manchester that takeaway management zones with those stringencies would lead to exposure to 42.6 (95% CI 38.7, 46.5) and 56.8 (95% CI 51.6, 61.9) fewer takeaways per person, relative to business as usual (Table S1).

**Table 2:** Estimated difference in mean number of takeaways an adult is exposed to in 2040 due to the intervention compared to business-as-usual

Local authority	Baseline exposure in 2018^a^	Estimated exposure by 2040 with no takeaway management zones	Mean difference in outlet exposure per person compared to business-as-usual scenario
Wandsworth	73.5	98.6	− 12.6 (− 9.51, − 15.6)
Manchester	91.4	148.2	− 28.4 (− 25.8, − 31.0)
Sheffield	74.9	117.7	− 21.4 (− 17.3, − 25.5)
Blackburn with Darwen	66.6	98.0	− 15.7 (− 9.43, − 22.0)
North Somerset	18.6	26.8	− 4.09 (− 3.50, − 4.69)
Fenland	17.7	24.1	− 3.20 (− 1.98, − 4.43)

Upper and lower confidence intervals are indicated in brackets

Trajectories of takeaway growth were assumed to increase until 2031 and then stabilise between 2031 and 2040

The intervention was based on a realistic scenario where new takeaway growth reduces by 50% each year following the intervention

^a^Estimated outlet exposure (from home, work and commuting) in 2018

### Changes in mean BMI after takeaway management zone implementation

Realistic implementation was associated with an estimated mean per person reduction in BMI that was greatest in Manchester (0.68 kg/m^2^; 95% CI 0.62, 0.75) and lowest in Fenland (0.08 kg/m^2^; 95% CI 0.05, 0.11) in 2031 compared to business-as-usual and that was overall greater in more urban LAs (Table 3). These patterns were consistent, but effects were stronger under an optimistic implementation scenario, and stronger still under perfect implementation, where in Manchester the intervention would result in BMI of 1.03 kg/m^2^ (95% CI 0.93, 1.25) and 1.37 kg/m^2^ (95% CI 1.24, 1.49) lower respectively, relative to business as usual (Table S2).

**Table 3:** Change in mean BMI in the adult population (2018 to 2040) in six specified local authorities, following implementation of takeaway management zones in 2018

Local authority	Baseline obesity level (%)^a^	Estimated change in BMI (kg/m^2^)
Wandsworth	14.4	− 0.30 (− 0.23, − 0.38)
Manchester	25.4	− 0.68 (− 0.62, − 0.75)
Sheffield	25.3	− 0.52 (− 0.42, − 0.61)
Blackburn with Darwen	23.0	− 0.38 (− 0.23, − 0.53)
North Somerset	23.0	− 0.10 (− 0.08, − 0.11)
Fenland	40.1	− 0.08 (− 0.05, − 0.11)

Trajectories of BMI were assumed to change until 2031 and then stabilise between 2031 and 2040

The intervention was based on a realistic scenario where new takeaway growth reduces by 50% each year following the intervention

^a^Percentage of adults aged 18 + who are living with obesity

### Change in prevalence of obesity, QALYs and healthcare cost savings to 2040

We estimated reductions in obesity prevalence for all LAs, compared to business as usual. In males, percentage point (PP) reductions in obesity prevalence ranged from 2.3PP (95% UI 2.9, 1.7) in Manchester to 1.5PP (95% UI 1.9, 1.1) in Wandsworth (Table 4). In females, these reductions ranged from 1.9PP (95% UI 2.4, 1.4) in Manchester and Sheffield to 1.5PP (95% UI 1.9, 1.2) in Wandsworth. Our models also estimated gains in total QALYs for all LAs, which ranged from a gain of 249 QALYs per 100,000 population for adults living in Wandsworth, to a gain of 194 QALYS per 100,00 adults living in North Somerset. In terms of healthcare cost savings, these ranged from £2.02 million saved per 100,000 adults in Wandsworth to £1.65 million saved per 100,000 adults living in North Somerset over the 22-year period. In a sensitivity analysis, perfect implementation (i.e. no new takeaways being allowed to open after policy adoption) resulted in healthcare cost savings, QALYs, and changes in prevalence of obesity being approximately twice that observed under a realistic implementation scenario in the main analysis (Table S3).

**Table 4:** Impact of the intervention on quality adjusted life years (QALYs), healthcare cost savings, and obesity prevalence in the adult population (2018 to 2040) in six specified local authorities

	Total QALYs gained			Healthcare cost saving^a,b^ (£ in millions)			Percentage point reduction in obesity prevalence	
	Males	Females	QALYs gained/100,000 population	Males	Females	Savings per 100, 000 population	Males	Females
Wandsworth	282 (204, 367)	231 (169, 300)	249.19	1.81 (1.26, 2.47)	2.36 (1.61, 3.35)	2.02 (1.39, 2.83)	− 1.5 (− 1.9, − 1.1)	− 1.5 (− 1.9, − 1.2)
Manchester	425 (317, 546)	270 (202, 343)	240.70	2.62 (1.88, 3.53)	2.82 (1.99, 3.92)	1.88 (1.34, 2.58)	− 2.3 (− 2.9, − 1.7)	− 1.9 (− 2.4, − 1.4)
Sheffield	344 (257, 437)	252 (189, 321)	205.00	2.22 (1.61, 2.97)	2.73 (1.93,3.75)	1.70 (1.22, 2.31)	− 2.2 (− 2.8, − 1.6)	− 1.9 (− 2.4, − 1.4)
Blackburn with Darwen	101 (76, 128)	75 (56, 95)	231.88	0.64 (0.45, 0.86)	0.80 (0.56, 1.12)	1.90 (1.33, 2.61)	− 1.9 (− 2.4, − 1.5)	− 1.8 (− 2.2, − 1.3)
North Somerset	284 (213, 363)	248 (185, 315)	193.64	1.93 (1.39, 2.59)	2.59 (1.82, 3.59)	1.65 (1.17, 2.25)	− 1.6 (− 2.0, − 1.2)	− 1.7 (− 2.0, − 1.2)
Fenland	61.2 (45.7, 78.4)	51.5 (38.6, 65.5)	221.57	0.40 (0.29, 0.54)	0.50 (0.35, 0.69)	1.77 ( 1.26, 2.42)	− 1.9 (− 2.4, − 1.4)	− 1.7 (− 2.1, − 1.3)

^a^Based on the following conditions: type II diabetes, ischemic heart disease, hypertensive heart disease, stroke, atrial fibrillation and flutter, colon and rectal cancer, oesophageal cancer, breast cancer (females only), asthma, low back pain, hip and knee arthritis

^b^Healthcare costs and population health are discounted as per NICE recommendations for public health interventions

### Change in incident cases of disease to 2040

The largest estimated reductions in cases of disease were for type II diabetes, with an estimated reduction of 1013 (95% UI 1285, 735) male and 837 (95% UI 1048, 634) female cases per 100,000 population, by 2040, in Blackburn with Darwen (Table 5). Reductions in all forms of cardiovascular disease were also observed, with reductions in IHD (e.g. Blackburn with Darwen, males: 153 cases/100,000 population, 95% UI 192, 117) and atrial fibrillation (e.g. Blackburn with Darwen, males: 73 cases/100,000 population, 95% UI 102, 48) strongest in all LAs, and consistently more pronounced in males. Improvements for respiratory health, with marked reductions in asthma, particularly for females (e.g. Blackburn with Darwen: 402 cases/100,000 population, 95% UI 603, 220), were also observed. Smaller reductions were estimated for oesophageal, breast, and colon and rectum cancers across all LAs. Of all cancers, case reductions were greatest for breast cancer. In terms of impacts on musculoskeletal disease, reductions were estimated for low back pain and more so for females than males (e.g. Blackburn with Darwen: 326 cases/100,000 population, 95% UI 644, 17). Small increases in incidence rates for hip and knee osteoarthritis were consistently estimated for both sexes in all LAs. In sensitivity analysis, perfect implementation resulted in an almost doubling of reductions, in disease incidence across all LAs, relative to realistic implementation (Table S4).

**Table 5:** Change in incident cases of disease/100,000 adult population (2018 to 2040), in six specified local authorities

	Blackburn with Darwen	Fenland	Manchester	Sheffield	North Somerset	Wandsworth
**Males**						
** Metabolic**						
Type II diabetes	− 1013 (− 1285, − 753)	− 995 (− 1262, − 740)	− 964 (− 1565, − 870)	− 803 (− 1023, − 594)	− 804 (− 1018, − 600)	− 1206 (− 1565, − 870)
**Cardiovascular disease**						
Ischaemic heart disease	− 153 (− 192, − 117)	− 105 (− 131, − 80.6)	− 124 (− 157, − 94.1)	− 118 (− 149, − 90.0)	− 91.1 (− 114, − 70.3)	− 99.2 (− 125, − 75.3)
Hypertensive heart disease	− 8.16 (− 13.5, − 3.22)	− 8.32 (− 13.6, − 3.45)	− 6.89 (− 11.9, − 2.13)	− 7.91 (− 13.1, − 3.09)	− 8.42 (− 13.7, − 3.59)	− 6.73 (− 11.9, − 2.03)
Stroke	− 9.32 (− 12.5, − 6.41)	− 15.9 (− 21.1, − 11.0)	− 21.1 (− 28.3, − 14.5)	− 17.3 (− 23.3, − 11.9)	− 15.2 (− 20.1, − 10.5)	− 18.9 (− 25.3, − 12.9)
Atrial fibrillation and flutter	− 72.5 (− 102, − 47.8)	− 61.7 (− 86.6, − 40.7)	− 60.6 (− 85.5, − 39.8)	− 59.7 (− 83.8, − 39.4)	− 57.3 (− 80.3, − 4.17) −	− 62 (− 87.7 − 40.6)
**Cancer**						
Colon and rectum cancer Oesophageal	− 1.05 (− 1.57, − 0.52) − 0.03 (− 0.05, − 0.03)	− 1.58 (− 2.77, − 0.79) − 3.16 (− 4.35, − 1.98)	− 1.59 (− 2.41, − 0.83) − 3.28 (− 4.54, − 2.24)	− 1.76 (− 2.64, − 0.93) − 2.94 (− 4.04, − 2.02)	− 1.22 (− 1.79, − 0.69) − 3.58 (− 5.00, − 2.44)	− 0.91. (− 1.31, − 0.50) < 0.01 (0.01, 0.01)
**Respiratory**						
Asthma	− 196 (− 293, − 107)	− 187 (− 283, − 101)	− 192 (− 288, − 105)	− 169 (− 252, − 93.4)	− 182 (− 278, − 97.2)	− 238 (− 366, − 125)
**Musculo-skeletal**						
Low back pain	− 272 (− 532, − 21.0)	− 278 (− 556, − 5.03)	− 332 (− 650, − 29.0)	− 277 (− 534, − 31.6)	− 332 (− 676, 1.58)	− 249 (− 534, 22.2)
Hip osteoarthritis Knee osteoarthritis	0.52 (0.52, 0.79) 2.62 (2.10, 2.62)	0.40 (0.40, − 0.40) 1.98 (1.58, 2.37)	0.6 (0.47, 0.73) 2.74 (2.20, 3.34)	0.55 (0.41, 0.62) 2.41 (1.92, 2.96)	0.15 (0.15, 0.22) 0.97 (0.75, 1.19)	0.30 (0.20, − 0.81) 1.21 (0.91, 1.51)
**Females**						
** Metabolic**						
Type II diabetes	− 837 (− 1048, − 634)	− 987 (− 1228, − 754)	− 725 (− 911, − 546)	− 677 (− 847, − 513)	− 801 (− 997, − 612)	− 879 (− 1116, − 648)
**Cardiovascular disease**						
Ischaemic heart disease	− 49.8 (− 62.3, − 38.3)	− 39.2 (− 48.9, − 30.2)	− 41.8 (− 52.7, − 31.8)	− 41.1 (− 51.7, − 31.4)	− 34.4 (− 42.8, − 26.6)	− 31.4 (− 39.7, − 23.9)
Hypertensive heart disease	− 5.47 (− 8.72, − 2.38)	− 5.92 (− 9.18, − 2.77)	− 4.68 (− 7.52, − 1.86)	− 5.37 (− 8.40, − 2.34)	− 6.18 (− 9.73, − 2.91)	− 4.40 (− 7.40, − 1.59)
Stroke	− 19.0 (− 25.6, − 13.5)	− 15.2 (− 20.4, − 10.8)	− 20.1 (− 27.2, − 13.9)	− 16.7 (− 22.5, − 11.6)	− 15.3 (− 20.4, − 10.9)	− 15.2 (− 20.7, − 10.5)
Atrial fibrillation and flutter	− 32.7 (− 45.9, − 21.6)	− 30.9 (− 43.3, − 20.5)	− 30.9 (− 43.3, − 20.5)	− 27.2 (− 38.1, − 18.0)	− 31.8 (− 44.6, − 21.1)	− 24.1 (− 34.1, − 15.8)
**Cancer**						
Colon and rectum cancer Oesophageal Breast cancer	− 1.02 (− 1.50, − 0.57) < 0.01 (0.01, 0.01) − 6.43 (− 8.63, − 4.49)	− 0.78 (− 1.56, − 0.39) − 0.78 (− 0.78, − 0.39) − 6.65 (− 8.99, − 4.69)	− 0.95 (− 1.40, − 0.53) < 0.01 (0.01, 0.01) − 6.56 (− 8.82, − 4.58)	− 0.96 (− 1.45, − 0.55) − 0.48 (− 0.69, − 0.34) − 6.61 (− 8.88, − 4.61)	− 1.14 (− 1.71, − 0.64) < 0.01 (0.01, 0.01) − 6.68 (− 8.95, − 4.69)	− 0.67 (− 1.03, − 0.37) < 0.01 (0.01, 0.01) − 6.28 (− 8.43, − 4.40)
**Respiratory**						
Asthma	− 402 (− 603, − 220)	− 327 (− 490, − 179)	− 361 (− 542, − 199)	− 325 (− 485, − 180)	− 318 (− 483, − 171)	− 444 (− 681, − 235)
**Musculo-skeletal**						
Low back pain	− 326 (− 644, 16.5)	− 312 (− 613, 16.7)	− 325 (− 638, − 23.8)	− 319 (− 618, 31.2)	− 316 (− 613, 1.51)	− 318 (− 666, 11.3)
Hip osteoarthritis Knee osteoarthritis	0.26 (0.26, 0.26) 1.06 (0.79, 1.32)	004 (0.03, 0.05) 0.78 (0.78, 1.17)	0.22 (0.14, 0.22) 1.08 (0.86, 1.37)	0.14 (0.14, 0.21) 0.96 (0.76, 1.17)	0.14 (0.14, 0.21) 0.78 (0.78, 1.17)	0.09 (0.09, 0.09) 0.47 (0.37, 0.66)

## Discussion

### Summary of findings

Our findings suggest that takeaway management zones around schools could make a substantive contribution to improving adult health and associated healthcare costs. We estimated that this intervention would reduce prevalence of obesity by 1.5 to 2.3 percentage points by 2031, leading to improvements in BMI-related health outcomes to 2040. These estimates were forecast to result in reductions in incidence of a range of diseases, including type II diabetes, cardiovascular diseases, and asthma. Estimated healthcare cost savings and gains in QALYs were similar in magnitude across LAs, with healthcare savings ranging between £1.65 and £2.02 million per 100,000 population, and gains in QALYs ranging from between 194 and 249 QALYs gained/100,000 population in North Somerset and Wandsworth, respectively. We also found that more stringent implementation of the policy, in alternate optimised or perfect scenarios, would result in even greater population health benefits.

### Comparison with other studies

This is the first study attempting to estimate health impacts of takeaway management zones, making it challenging to make direct comparisons with other studies. However, reductions in obesity prevalence in relation to takeaway management zones were consistent across LAs and in line with a number of other studies that have found a relationship between higher exposure to takeaways and increased BMI or risk of obesity in adults [11–13]. Meaningful reductions were estimated for future incidence of 12 obesity-related diseases to 2040 across all LAs irrespective of rural–urban classification. The most pronounced reductions, in all LAs, were in incidence of type II diabetes, which in males ranged from reductions of 803 cases/100,000 population in Sheffield to 1206 cases/100,000 population in Wandsworth. Consistent with this finding, previous studies have shown a positive association between residential takeaway exposure and prevalence of type II diabetes [7, 9]. This is an important finding because aside from older age, type II diabetes incurs the biggest financial cost of any single disease to the national healthcare service, accounting for 8% of secondary care costs and occupying 17% of hospital day-beds [42]. Our estimates also showed substantial reductions in incidence of cardiovascular diseases in response to adoption of management zones. The largest reductions in incident cases were seen in ischaemic heart disease (IHD), with smaller reductions in stroke and hypertensive heart disease. Consistent with this finding, a recent systematic review highlighted evidence of a relationship between takeaway exposure and cardiovascular disease risk [43]. Furthermore, another study found that incidence of CVD and to a lesser extent stroke was also higher in adults exposed to more takeaways, which mirrors our observations [8]. Our model also estimated meaningful reductions in the incidence of some cancers, asthma, and low back pain. While research on the link between takeaway exposure and these conditions is lacking, each has been found to be associated with living with obesity [44–46].

It is challenging to benchmark our modelled estimates of health benefits associated with management zones against those from other public health interventions. Direct comparisons are problematic due to differences in study populations, baseline levels of disease risk, type of cohort (open or closed), duration of study, and outcomes studied as well as differences in the type, intensity, and reach of the intervention being studied. However, we are aware of the modelled impacts of two public health interventions that yield similar health benefits to our estimates and suggest our findings are plausible [47]. Gains in QALYs over 10 years as a result of reformulating food in England such that it meets the 2017 Food Standards Agency salt targets were broadly comparable to our own. For example, 126 QALYs/100,000 population gained in men aged 70–74 years was estimated, compared to gains in our study that ranged from 194 to 249 in adults aged 26–65 years. Elsewhere, another intervention was the nationwide expansion of Birmingham’s ‘Be Active’ scheme, which provides free access to council leisure centres at certain times. This intervention was estimated to reduce the incidence rate of ischemic heart disease by 144 and 61/100,000 population in males and females, respectively. These gains are comparable with those from our study, where males, for example, saw a reduction in incidence between 91/100,000 population (males and females combined) in North Somerset and 153/100,000 population in Blackburn, respectively.

### Interpretation of findings

Recent data from the Health Survey for England suggested that approximately 26% of adults are obese, with the highest prevalence in age-groups 45–74 years [48]. This suggests that adults in this age group may be an important group to target, especially given the relationship between obesity and disability and chronic disease in older adults [49]. However, while the significant reductions in obesity prevalence estimated by our models are encouraging (e.g. 1.9 PP in females in Sheffield), they also illustrate the need for a broader set of diet-related interventions to further reduce prevalence of obesity. Many public health interventions are cost saving [50], and while the financial costs of the implementation of takeaway management zones were not included in our study and should be integrated into future analyses, healthcare savings were estimated to range from £1.65 million per 100,000 population in North Somerset to £2.02 million per 100,000 population in Wandsworth by 2040. If sustained over a period of 22 years, our modelling also showed that takeaway management zones could add between 612 (Fenland) and 425 (Manchester) QALYs for males alone, suggesting that the intervention has the potential to make meaningful improvements to the quality of life of whole populations. Our models also estimated slight increases in incidence of knee and hip osteoarthritis. While BMI is associated with osteoarthritis [51], this finding can be explained by the higher proportion of older adults surviving in the population because of the intervention [52]. While our findings estimate larger BMI reductions in more urban LAs, our modelling of health impacts does not mirror this difference between urban and rural areas with incidence of non-communicable diseases and change in obesity prevalence, healthcare savings, and QALYs per person. This finding may reflect differences in the demographics of selected LAs including baseline obesity levels, deprivation and age, which are risk factors for poor health.

### Study limitations

#### Limitations: forecasting model of takeaway growth

Our study makes use of unique forecasts of long-term population exposure to takeaways in the absence of intervention, in six different types of LAs, based on continuation of pre-existing trends in takeaway growth. As the intervention can only stop future growth in takeaway retail, the benefits of the intervention are contingent on this continued growth (in the absence of intervention), but this is inherently uncertain. For example, to what extent will growth in numbers of physical premises continue if online takeaway delivery use continues to rise. Further details on the limitations of this forecasting method have been published previously [53]. There is also uncertainty around the impact of local differences in implementation on long-term health outcomes. To address this, in addition to a core scenario based on recent estimates of real-world impact [28], we also provided estimates based on alternative scenarios.

#### Limitations: exposure to takeaway outlets and BMI

We were unable to capture the amount of time spent at work, home and commuting. However, we draw on existing evidence that higher levels of exposure to takeaway outlets in these three domains is associated with higher BMI in the population regardless of the time spent in each [11].

This study builds on previous evidence suggesting an association between exposure to takeaway outlets and BMI. A linear relationship between takeaway outlet exposure and BMI was reported using data from an observational, cross-sectional analysis of Fenland Study data in the UK, and the magnitude of this association underpinned our modelling [11]. Other studies using more robust causal methods including experimental and longitudinal studies have not been conducted in the UK. This is important because where such studies have been conducted elsewhere around the world, this relationship has been found to be inconsistent across studies, suggesting potential differences between countries. For example, a longitudinal study of adults living in the Netherlands found that increases in fast-food outlet density within 1 km of the home was associated with increases in BMI up to 4 years later [15]. However, a US study found no consistent relationship between access to fast-food establishments and BMI [19].

#### Limitations: generalisability

We make use of forecasts in six different types of LA to promote some degree of generalisability across the urban–rural spectrum. However, our findings are unlikely to be generalisable to all LAs in England. Our findings are not readily generalisable to children. In this study, we focussed on the adult population, primarily because previously published associations between takeaway exposure and BMI were in UK adults [11]. Also, because takeaway management zones cover a wide geographical area, it is reasonable to assume they will also impact adults. Moreover, the geographic and social determinants of takeaway consumption in children may be different, and this should be the subject of future research. While observational studies in children show an association between takeaway consumption and energy intake, no corresponding association between takeaway consumption and body weight has been observed, perhaps because energy demands tend to be higher for growth and development [54]. Evidence on the relationship between exposure to takeaways and body weight in older populations is also currently lacking; thus, our models did not include adults who were aged 65 years and over at study baseline. However, a study using data from the UK National Diet and Nutrition Survey found that adults aged 70 years and over were one fifth as likely to eat takeaway meals at home compared to young adults aged 19–29 years. This supports the idea that dietary behaviours are subject to change over the life course [39], necessitating further modelling of intervention impacts in this older age group.

#### Limitations: PRIMEtime modelling

The PRIMEtime model excludes some important diseases associated with BMI, including depression and dementia, potentially leading to our results being an underestimation of effect sizes for savings in healthcare costs and QALYs [55, 56]. In choosing to model a closed cohort, we will have potentially further underestimated health and healthcare cost savings. BMI is also positively linked to need for social care provision [57], however, we have not modelled social care costs. In the UK, social care costs (in contrast to healthcare costs) are borne by the local authority, and so the returns to the body that bears the risks and costs of the intervention are not quantified here.

PRIMEtime assumes remission from disease to be zero, which may affect the estimate of cases prevented by the modelling. As the prevalence of disease is dynamically modelled over age in the model, the slightly decreased prevalence rate could lead to an overestimate of the impact of the intervention on incidence rate (via the Population Impact Fraction). However, as the number of cases of cancers is much smaller than those of other diseases, it does not appear this effect could be meaningfully biasing results.

#### Limitations: online food delivery

We were unable to account for the impact of online food delivery services (e.g. Just Eat, Deliveroo), which may attenuate the relationship between takeaway management zones and health. These fast-growing services are likely to increase the availability of takeaway food, which the intervention was designed to reduce, thereby reducing its impact. In one UK study, online food delivery services were used at least once per week by approximately 15% of adults in 2018 [58], and there is evidence that access is unequal between urban and rural areas [59]. From 2020 to 2022, access to online delivery takeaways was found to have increased by 10% for those living in the most deprived areas of England [60]. Adults living in the UK who have access to the greatest number of takeaways online were also found to have the greatest odds of using online food delivery services [58, 61]. Future research should consider the possibility that place-based interventions such as management zones may to some extent be undermined by new modes of takeaway food purchasing.

### Policy implications and future directions

A lack of evidence of health benefits associated with the adoption of takeaway management zones around schools has been cited as a barrier to policy adoption and effective implementation [29, 30]. Building on recent studies that have observed the retail impacts of policy adoption [28, 62], our modelling work now provides evidence on the population health impacts that could be achieved through the adoption and (even imperfect) implementation of takeaway management zones around schools. We also showed how stricter, perfect, or even optimised implementation (preventing takeaway growth by 100% and 75%, respectively) would result in even greater health to 2040. Local decision makers should therefore remain diligent in the strict implementation of takeaway management zones if optimum population health is to be achieved.

In addition to a range of health benefits, we also modelled health care expenditure benefits associated with the adoption of takeaway management zones around schools, which were achieved through a reduction in healthcare costs. Although these economic benefits may not accrue locally, these cost savings are important evidence for those working in LAs who seek to understand the wider health benefits of management zones [63]. It is still possible, however, as argued by inspectors from the national planning inspectorate, that management zones could be detrimental to the economy, through denying business growth and curtailing employment opportunities [30]. As public health interventions are liable to legal challenge under the principle of proportionality, future work should include a full economic analysis, considering both health and social care costs and benefits, alongside these other economic considerations. Future studies should also account for the continued emergence and growth of online food delivery platforms, which could diminish the health impacts of this intervention.

## Conclusions

In response to a realistic intervention scenario and across a range of different types of LAs, we found meaningful reductions in population-level BMI and obesity prevalence, as well as reductions in a variety of associated non-communicable diseases including incidence of type II diabetes, cardiovascular diseases, cancers, asthma, and low back pain, to the year 2040. We also found associated health-related benefits including gains in QALYs and savings in healthcare costs. Takeaway management zones around schools may be an effective population-level intervention to improve diet-related health in adults in the UK [64].

## Supplementary Information

Additional file 1. Tables S1-S4. Table S1. Estimated difference in mean number of takeaways a person is exposed to due to the intervention compared to business as usual. Table S2. Change in mean BMI for the adults aged 25–64 years across 6 specified local authorities in England, by 2031, following implementation of takeaway exclusion zones in 2018. Table S3. Impact of the intervention, on QALYs, health care costs and change in obesity prevalence in the adult population from 2018–2040 in specified local authorities. Table S4. Change in incident cases of disease per 100,000 adult population (2018 to 2040), in specified local authorities as a result of the intervention, assuming 100% stringency.

## Data Availability

Derived data on takeaway outlet growth projections is available on request from the corresponding author. The data used to support the PRIMEtime model, some with licenced agreements from relevant data archives (e.g. Health Survey for England data), are available in the public domain.

## Abbreviations

BMI: Body mass index
IHD: Ischaemic heart disease
LA: Local authority
NICE: National Institute for Health and Care Excellence
PP: Percentage points
QALY: Quality-adjusted life years
UI: Uncertainty interval

## References

[1] Bowman SA, Vinyard BT. Fast Food Consumption of U.S. Adults: impact on energy and nutrient intakes and overweight status. J Am Coll Nutr. 2004;23:163–8. 15047683 10.1080/07315724.2004.10719357

[2] Lachat C, Nago E, Verstraeten R, Roberfroid D, Van Camp J, Kolsteren P. Eating out of home and its association with dietary intake: a systematic review of the evidence. Obes Rev. 2012;13:329–46. 22106948 10.1111/j.1467-789X.2011.00953.x

[3] Goffe L, Rushton S, White M, Adamson A, Adams J. Relationship between mean daily energy intake and frequency of consumption of out-of-home meals in the UK National Diet and Nutrition Survey. Int J Behav Nutr Phys Act. 2017;14:1–11. 28938893 10.1186/s12966-017-0589-5 PMC5610411

[4] Schröder H, Fito M, Covas MI. Association of fast food consumption with energy intake, diet quality, body mass index and the risk of obesity in a representative Mediterranean population. Br J Nutr. 2007;98:1274–80. 17625027 10.1017/S0007114507781436

[5] Duffey KJ, Gordon-Larsen P, Steffen LM, Jacobs DR, Popkin BM. Regular consumption from fast food establishments relative to other restaurants is differentially associated with metabolic outcomes in young adults. J Nutr. 2009;139:2113–8. 19776183 10.3945/jn.109.109520 PMC2762152

[6] Prentice AM, Jebb SA. Fast foods, energy density and obesity: a possible mechanistic link. Obes Rev. 2003;4:187–94. 14649369 10.1046/j.1467-789x.2003.00117.x

[7] Ntarladima AM, Karssenberg D, Poelman M, et al. Associations between the fast-food environment and diabetes prevalence in the Netherlands: a cross-sectional study. Lancet Planet Health. 2022;6:e29-39. 34998457 10.1016/S2542-5196(21)00298-9

[8] Poelman M, Strak M, Schmitz O, et al. Relations between the residential fast-food environment and the individual risk of cardiovascular diseases in The Netherlands: a nationwide follow-up study. Eur J Prev Cardiol. 2018;25:1397–405. 29688759 10.1177/2047487318769458 PMC6130123

[9] Sarkar C, Webster C, Gallacher J. Are exposures to ready-to-eat food environments associated with type 2 diabetes? A cross-sectional study of 347 551 UK Biobank adult participants. Lancet Planet Health. 2018;2:e438–50. 30318101 10.1016/S2542-5196(18)30208-0

[10] Glanz K,Sallis JF, Saelens BE, Frank LD. Healthy nutrition environments: concepts and measures. Am J Health Promot. 2005;19:330–3. 15895534 10.4278/0890-1171-19.5.330

[11] Burgoine T, Forouhi NG, Griffin SJ, Wareham NJ, Monsivais P. Associations between exposure to takeaway food outlets, takeaway food consumption, and body weight in Cambridgeshire, UK: population based, cross sectional study. BMJ (Online). 2014;348:1–10. 10.1136/bmj.g1464 PMC3953373 24625460

[12] van Erpecum CPL, van Zon SKR, Bültmann U, Smidt N. The association between the presence of fast-food outlets and BMI: the role of neighbourhood socio-economic status, healthy food outlets, and dietary factors. *BMC Public Health* 2022; 22. 10.1186/S12889-022-13826-1. 10.1186/s12889-022-13826-1 PMC9331587 35897088

[13] Burgoine T, Sarkar C, Webster CJ, Monsivais P. Examining the interaction of fast-food outlet exposure and income on diet and obesity: Evidence from 51,361 UK Biobank participants. Int J Behav Nutr Phys Act. 2018;15:1–12. 30041671 10.1186/s12966-018-0699-8 PMC6497220

[14] Athens JK, Duncan D, Elbel B. Proximity to fast food outlets and supermarkets as predictors of fast food dining frequency. *J Acad Nutr Diet* 2017; **116**. 10.1016/j.jand.2015.12.022 PMC4967005 26923712

[15] van Erpecum CPL, van Zon SKR, Bültmann U, Smidt N. Effects of changes in residential fast-food outlet exposure on body mass index change: longitudinal evidence from 92,211 Lifelines participants. *International Journal of Behavioral Nutrition and Physical Activity* 2024; 21. 10.1186/s12966-024-01577-8. 10.1186/s12966-024-01577-8 PMC10941418 38486265

[16] Fraser L, Edwards K. The association between the geography of fast food outlets and childhood obesity rates in Leeds. UK Health Place. 2010;16:1124–8. 20691630 10.1016/j.healthplace.2010.07.003

[17] Mackenbach JD, Charreire H, Glonti K, et al. Exploring the relation of spatial access to fast food outlets with body weight: a mediation analysis. Environ Behav. 2019;51:401–30.

[18] Lam TM, Vaartjes I, Grobbee DE, Karssenberg D, Lakerveld J. Associations between the built environment and obesity: an umbrella review. Int J Health Geogr. 2021; **20**. 10.1186/s12942-021-00260-6. 10.1186/s12942-021-00260-6 PMC7852132 33526041

[19] Block JP, Christakis NA, James O’malley A, Subramanian S V. Original contribution proximity to food establishments and body mass index in the Framingham Heart Study offspring cohort over 30 years. 2011. 10.1093/aje/kwr244. 10.1093/aje/kwr244 PMC3208142 21965186

[20] Dekker LH, Rijnks RH, Strijker D, Navis GJ. A spatial analysis of dietary patterns in a large representative population in the north of The Netherlands - the Lifelines cohort study. Int J Behav Nutr Phys Act. 2017;14:166. 29212502 10.1186/s12966-017-0622-8 PMC5719934

[21] Zijlema WL, Klijs B, Stolk RP, Rosmalen JGM. (Un)healthy in the city: respiratory, cardiometabolic and mental health associated with urbanity. *PLoS One* 2015; 10. 10.1371/journal.pone.0143910. 10.1371/journal.pone.0143910 PMC4667966 26630577

[22] van Erpecum CPL, van Zon SKR, Bültmann U, Smidt N. The association between fast-food outlet proximity and density and Body Mass Index: findings from 147,027 Lifelines Cohort Study participants. Prev Med (Baltim). 2022;155: 106915. 10.1016/j.ypmed.2021.106915 34922992

[23] Takeaway & Fast-Food Restaurants in the UK - Number of Businesses. *IBISWorld* 2023.

[24] The Takeaway Economy Report. Centre for Economics and Business Research. 2017.

[25] Day P, Pearce J. Obesity-promoting food environments and the spatial clustering of food outlets around schools. Am J Prev Med. 2011;40:113–21. 21238858 10.1016/j.amepre.2010.10.018

[26] Blow J, Gregg R, Davies IG, Patel S. Type and density of independent takeaway outlets: a geographical mapping study in a low socioeconomic ward. Manchester BMJ Open. 2019;9:1–7. 10.1136/bmjopen-2018-023554 PMC6661625 31340954

[27] Rahilly J, Williams A, Chang M, *et al.* Changes in the number and outcome of takeaway food outlet planning applications in response to adoption of exclusion zones around schools in England: a time series analysis. *Health Place* 2023. 10.1016/j.healthplace.2024.103237 38564989

[28] Rahilly J, Amies-cull B, Chang M, *et al.* Changes in the number of new takeaway food outlets associated with adoption of management zones around schools: a natural experimental evaluation in England. *SSM Popul Health* 2024:101646. 10.1016/j.ssmph.2024.101646 PMC11033196 38650739

[29] Keeble M, Burgoine T, White M, Summerbell C, Cummins S, Adams J. Planning and Public Health professionals’ experiences of using the planning system to regulate hot food takeaway outlets in England: a qualitative study. Health Place. 2021;67:102305. 33526206 10.1016/j.healthplace.2020.102305 PMC7613884

[30] O’Malley CL, Lake AA, Townshend TG, Moore HJ. Exploring the fast food and planning appeals system in England and Wales: decisions made by the Planning Inspectorate (PINS). Perspect Public Health. 2021;141:269–78. 32580644 10.1177/1757913920924424 PMC8392781

[31] Garde A. Law, healthy diets and obesity prevention. 2015.

[32] Metcalf CJE, Edmunds WJ, Lessler J. Six challenges in modelling for public health policy. Epidemics. 2015;10:93–6. 25843392 10.1016/j.epidem.2014.08.008

[33] Cobiac L, Law C, Scarborough P. PRIMEtime: an epidemiological model for informing diet and obesity policy. *medRxiv* 2024.

[34] Keeble M, Burgoine T, White M, Summerbell C, Cummins S, Adams J. How does local government use the planning system to regulate hot food takeaway outlets? A census of current practice in England using document review. Health Place. 2019;57:171–8. 31055107 10.1016/j.healthplace.2019.03.010 PMC6686733

[35] Liu B, Mytton O, Rahilly J, *et al.* Development of an approach to forecast future takeaway outlet growth around schools and population exposure in England. *International Journal of Health Geographics In Press* 2024. 10.1186/s12942-024-00383-6 PMC11550555 39523305

[36] Burgoine T, Monsivais P, Sharp SJ, Forouhi NG, Wareham NJ. Independent and combined associations between fast-food outlet exposure and genetic risk for obesity: a population-based, cross-sectional study in the UK. BMC Med. 2021;19:1–9. 33588846 10.1186/s12916-021-01902-z PMC7885578

[37] Briggs ADM, Scarborough P, Wolstenholme J. Estimating comparable English healthcare costs for multiple diseases and unrelated future costs for use in health and public health economic modelling. 2018. 10.1371/journal.pone.0197257. 10.1371/journal.pone.0197257 PMC5967835 29795586

[38] NICE. Guide to the methods of technology appraisal. London: National Institute for Health andnCare Excellence. 2013. 27905712

[39] Adams J, Goffe L, Brown T, et al. Frequency and socio-demographic correlates of eating meals out and take-away meals at home: cross-sectional analysis of the UK national diet and nutrition survey, waves 1–4 (2008–12). Int J Behav Nutr Phys Act. 2015;12:1–9. 25889159 10.1186/s12966-015-0210-8 PMC4404110

[40] Lawes C, Vander Hoorn S, Law M, Elliott P. Comparative quantification of health risks: global and regional burden of disease attributable to selected major risk factors. 2004.

[41] Eliassen AH, Colditz GA, Rosner B, Willett WC, Hankinson SE. Adult weight change and risk of postmenopausal breast cancer. JAMA. 2006;296:193. 16835425 10.1001/jama.296.2.193

[42] Stedman M, Lunt M, Davies M, et al. Cost of hospital treatment of type 1 diabetes (T1DM) and type 2 diabetes (T2DM) compared to the non-diabetes population: a detailed economic evaluation. BMJ Open. 2020;10: e033231. 32376746 10.1136/bmjopen-2019-033231 PMC7223153

[43] Meijer P, Numans H, Lakerveld J. Associations between the neighbourhood food environment and cardiovascular disease: a systematic review. Eur J Prev Cardiol. 2023;30:1840–50. 37499177 10.1093/eurjpc/zwad252

[44] Pati S, Irfan W, Jameel A, Ahmed S, Shahid RK. Obesity and cancer: a current overview of epidemiology, pathogenesis, outcomes, and management. Cancers (Basel). 2023;15:1–21. 10.3390/cancers15020485 PMC9857053 36672434

[45] Peters U, Dixon AE, Forno E. Obesity and asthma. Journal of Allergy and Clinical Immunology. 2018;141:1169–79. 29627041 10.1016/j.jaci.2018.02.004 PMC5973542

[46] Su CA, Kusin DJ, Li SQ, Ahn UM, Ahn NU. The association between body mass index and the prevalence, severity, and frequency of low back pain. Spine (Phila Pa 1976). 2018;43:848–52. 29462069 10.1097/BRS.0000000000002601

[47] Briggs ADM, Wolstenholme J, Scarborough P. Estimating the cost-effectiveness of salt reformulation and increasing access to leisure centres in England, with PRIMEtime CE model validation using the AdViSHE tool. *BMC Health Serv Res* 2019; **19**. 10.1186/s12913-019-4292-x. 10.1186/s12913-019-4292-x PMC6631881 31307459

[48] House of Commons library. Obesity statistics. 2023.

[49] Samper-Ternent R, Al SS. Obesity in older adults: epidemiology and implications for disability and disease. Rev Clin Gerontol. 2012;22:10–34. 22345902 10.1017/s0959259811000190 PMC3278274

[50] Masters R, Anwar E, Collins B, Cookson R, Capewell S. Return on investment of public health interventions: a systematic review. J Epidemiol Community Health. 1978;2017(71):827–34. 10.1136/jech-2016-208141 PMC5537512 28356325

[51] Jiang L, Tian W, Wang Y, et al. Body mass index and susceptibility to knee osteoarthritis: a systematic review and meta-analysis. Joint Bone Spine. 2012;79:291–7. 21803633 10.1016/j.jbspin.2011.05.015

[52] Berrington de Gonzalez A, Hartge P, Cerhan JR, *et al.* Body-mass index and mortality among 1.46 million white adults. *N Engl J Med* 2010; **363**: 2211–9. 10.1056/NEJMoa1000367 PMC3066051 21121834

[53] Liu B, Mytton O, Rahilly J, *et al.* Development of an approach to forecast future takeaway outlet growth around schools and population exposure in England. 2024. 10.1186/s12942-024-00383-6 PMC11550555 39523305

[54] Dolton PJ, Tafesse W. Childhood obesity, is fast food exposure a factor? Econ Hum Biol. 2022;46: 101153. 35809404 10.1016/j.ehb.2022.101153

[55] De Wit LM, Van Straten A, Van Herten M, Penninx BW, Cuijpers P. Depression and body mass index, a u-shaped association. BMC Public Health. 2009;9:1–6. 19144098 10.1186/1471-2458-9-14 PMC2631467

[56] Kivimäki M, Luukkonen R, Batty GD, et al. Body mass index and risk of dementia: analysis of individual-level data from 1.3 million individuals. Alzheimer’s and Dementia. 2018;14:601–9. 29169013 10.1016/j.jalz.2017.09.016 PMC5948099

[57] Copley VR, Cavill N, Wolstenholme J, Fordham R, Rutter H. Estimating the variation in need for community-based social care by body mass index in England and associated cost: population-based cross-sectional study. BMC Public Health. 2017;17:1–11. 28830401 10.1186/s12889-017-4665-1 PMC5567467

[58] Keeble M, Adams J, Sacks G, et al. Use of online food delivery services to order food prepared away-from-home and associated sociodemographic characteristics: a cross-sectional, multi-country analysis. Int J Environ Res Public Health. 2020;17:1–17. 10.3390/ijerph17145190 PMC7400536 32709148

[59] Kalbus A, Ballatore A, Cornelsen L, Greener R, Cummins S. Associations between area deprivation and changes in the digital food environment during the COVID-19 pandemic: longitudinal analysis of three online food delivery platforms. Health Place. 2023;80: 102976. 36758447 10.1016/j.healthplace.2023.102976 PMC9899780

[60] Keeble M, Adams J, Burgoine T. Changes in online food access during the COVID-19 pandemic and associations with deprivation: longitudinal analysis. *JMIR Public Health Surveill* 2023; **9**. 10.2196/41822. 10.2196/41822 PMC10131934 36848236

[61] Keeble M, Adams J, Vanderlee L, Hammond D, Burgoine T. Associations between online food outlet access and online food delivery service use amongst adults in the UK: a cross-sectional analysis of linked data. BMC Public Health. 2021;21:1–12. 34719382 10.1186/s12889-021-11953-9 PMC8557109

[62] Brown H, Xiang H, Albani V, et al. No new fast-food outlets allowed! Evaluating the effect of planning policy on the local food environment in the North East of England. Soc Sci Med. 2022;306:115126. 35724588 10.1016/j.socscimed.2022.115126

[63] Local Government Association. Money Well Spent, Assessing the cost effectiveness and return on investment of public health interventions. 2013.

[64] Adams J, Mytton O, White M, Monsivais P. Why are some population interventions for diet and obesity more equitable and effective than others? The role of individual agency. PLoS Med. 2016;13:1–7. 10.1371/journal.pmed.1001990 PMC4821622 27046234

