# Supplementary material for: Health impacts of takeaway management zones around schools in six different local authorities across England: a public health modelling study using PRIMEtime
Source: BMC Med. 2024 Nov 19;22:545. doi: 10.1186/s12916-024-03739-8 (PMC11575031; doi:10.1186/s12916-024-03739-8)
Supplement: Supplementary file 1 — Additional file 1. Tables S1-S4. Table S1. Estimated difference in mean number of takeaways a person is exposed to due to the intervention compared to business as usual. Table S2. Change in mean BMI for the adults aged 25–64 years across 6 specified local authorities in England, by 2031, following implementation of takeaway exclusion zones in 2018. Table S3. Impact of the intervention, on QALYs, health care costs and change in obesity prevalence in the adult population from 2018–2040 in specified local authorities. Table S4. Change in incident cases of disease per 100,000 adult population (2018 to 2040), in specified local authorities as a result of the intervention, assuming 100% stringency. [file 12916_2024_3739_MOESM1_ESM.docx]

**Supplementary Tables**

Table S1. Estimated difference in mean number of takeaways a person is exposed to due to the intervention compared to business as usual.

Table S2. Change in mean BMI for the adults aged 25-64 years across 6 specified local authorities in England, by 2031, following implementation of takeaway exclusion zones in 2018.

Table S3. Impact of the intervention, on QALYs, health care costs and change in obesity prevalence in the adult population from 2018-2040 in specified local authorities.

Table S4. Change in incident cases of disease per 100,000 adult population (2018 to 2040), in specified local authorities as a result of the intervention, assuming 100% stringency

Table S1: Estimated difference^1^ in mean number of takeaways a person is exposed to due to the intervention^2^ compared to business as usual.

|  | Baseline  Exposure in 2018^3^ | Estimated difference in outlet exposure/person in 2031 compared to business as usual scenario | |
| --- | --- | --- | --- |
|  | | Optimistic^1^ | Perfect^1^ |
| Wandsworth | 73.5 | -18.8(-14.3, -23.4) | -25.1(-19.0, -31.2) |
| Manchester | 91.4 | -42.6(-38.7, 46.5) | -56.8(-51.6, -61.9) |
| Sheffield | 74.9 | -32.1(-26.0, -38.2) | -42.8(-34.7, -51.0) |
| Blackburn with Darwen | 66.6 | -23.6(-14.1, -33.0) | -31.4(-18.9, -44.0) |
| North Somerset | 18.6 | -6.14(-5.24, -7.04) | -8.19(-6.99, -9.38) |
| Fenland | 17.7 | -4.80(-2.96, 6.64) | -6.40(-4.00, -8.85) |

^1^Upper and Lower confidence intervals are indicated in brackets.

^2^The intervention here is based on an optimistic and perfect scenario where new takeaway growth reduces by 75% and 100% each year, respectively, following the intervention.

^3^Estimated Outlet exposure (from home, work and commuting) in 2018

|  |  | Estimated changes in BMI (kg/m^2^) | |
| --- | --- | --- | --- |
|  | Baseline Obesity levels (%)^2^ | Optimistic^3^ | Perfect^3^ |
| Wandsworth | 14.4 | -0.45(-0.34, -0.56) | -0.61(-0.46, -0.75) |
| Manchester | 25.4 | -1.03(-0.93, -1.25) | -1.37(-1.24, -1.49) |
| Sheffield | 25.3 | -0.77(-0.63, -0.92) | -1.03(-0.84, -1.23) |
| Blackburn with Darwen | 23.0 | -0.57(-0.34, -0.79) | -0.76(-0.45, -1.06) |
| North Somerset | 23.0 | -0.15(-0.13, -0.17) | -0.20(-0.17, -0.23) |
| Fenland | 40.1 | -0.12(-0.07, -0.16) | -0.15(-0.10, -0.21) |

Table S2. Change in mean BMI for the adults aged 25-64 years across 6 specified local authorities in England, by 2040^1^, following implementation of takeaway exclusion zones in 2018.

^1^ Trajectories of takeaway growth were assumed to increase until 2031 and then stabilise from 2031-2040.

^2^Percentage of adults aged 18 + who are living with obesity.

^3^ The intervention here is based on an optimistic and perfect scenario where new takeaway growth reduces by 75% and 100% each year, respectively, following the intervention

Table S3: Impact of the intervention, on QALYs, health care costs and change in obesity prevalence in the adult population from 2018-2040 in specified local authorities.

|  | Total QALYs | | Healthcare cost savings^1^ (£ in millions) | | Change in prevalence of obesity (PP) | |
| --- | --- | --- | --- | --- | --- | --- |
|  | Males | Females | Males | Females | Males | Females |
| Optimistic (75% stringency) | | | | | | |
| Wandsworth | 420 (309, 551) | 344 (253, 447) | 2.68(1.86, 3.71) | 3.49 (2.34, 4.93) | -2.2 (-1.7, -2.8) | -2.4 (-1.9, -3.0) |
| Manchester | 635 (478, 812) | 403 (302, 512) | 3.89 (2.75, 5.21) | 4.19 (2.89, 5.80) | -3.3 (-4.2, -2.5) | -2.9 (-3.7, -2.2) |
| Sheffield | 514 (387, 654) | 377(284, 480) | 3.31(4.41, 2.34) | 4.05(2.82,5.60) | -3.3 (-4.1, -2.5) | -2.9 (-3.7, -2.2) |
| Blackburn with Darwen | 151 (113, 192) | 111 (84, 143) | 0.95 (0.67, 1.27) | 1.2 (0.82, 1.66) | -2.9 (-3.7, -2.2) | -2.8 (-3.5, -2.1) |
| North Somerset | 424 (321, 542) | 370 (279, 470) | 2.86 (2.03, 3.82) | 3.84 (2.67, 5.31) | -2.3(-2.8, -1.8) | -2.5 (-3.1, -1.9) |
| Fenland | 91.5 (69.2, 117) | 77.0 (58.2, 97.5) | 0.59 (0.42,0.80) | 0.74 (0.52, 1.02) | -2.8 (-3.5, -2.2) | -2.7 (-2.1, -3.4) |
| Perfect (100% stringency) | | | | | | |
| Wandsworth | 550 (392, 733) | 450 (325, 592) | 3.50 (2.37, 4.96) | 4.56 (2.97, 6.53) | -2.9 (-2.3, -3.6) | -3.2 (-2.5, -4.0) |
| Manchester | 831 (609, 1077) | 527 (391, 674) | 5.09 (3.54, 7.07) | 5.49 (3.67, 7.68) | -4.4 (-5.4, -3.4) | -3.8 (-4.8, -3.0) |
| Sheffield | 673(502, 868) | 494 (368, 633) | 4.34 (3.04, 5.98) | 5.31 (3.58, 7.41) | -4.3 (-3.3, -5.3) | -3.9 (-3.0, -4.8) |
| Blackburn with Darwen | 198 (147, 256) | 146 (109, 188) | 1.24 (1.73, 0.86) | 1.56(2.19, 10.4) | -3.9 (-4.8, -3.0) | -3.6 (-4.5, -2.8) |
| North Somerset | 555 (412, 717) | 485 (361, 625) | 3.74 (2.62, 5.21) | 5.03 (3.37, 7.00) | -3.0(-3.7, -2.3) | -3.3(-4.0, -2.5) |
| Fenland | 120 (88, 155) | 120 (155, 88) | 0.78 (10.8, 0.55) | 0.78 (10.8, 0.55) | -3.7 (-4.4, -2.8) | -3.6 (-4.4, -2.8) |

|  | Blackburn with Darwen | Fenland | Manchester | Sheffield | North Somerset | Wandsworth |
| --- | --- | --- | --- | --- | --- | --- |
| **Males** | | | | | | |
| **Metabolic** |  |  |  |  |  |  |
| Type II diabetes | -1961 (-2503, -1452) | -1926 (-2453, -1428) | -1864 (-2396, -1354) | -1553(-1982, -1143) | -1556 (-1979, -1158) | -2327 (-3054, -1657) |
| **Cardiovascular disease** |  |  |  |  |  |  |
| Ischaemic heart disease | -301 (-373, -233) | -206 (-254, -159) | -244 (-306, -187) | -231 (-289, -179) | -179 (-220, -139) | -194 (-242, -142) |
| Hypertensive heart disease | -16.0 (-26.5, -5.77) | -16.2 (-26.9, -6.33) | -13.4 (23.6, -3.94) | -15.4 (-25.8, -5.50) | -16.4 (-27.1, -6.64) | -13.0 (-23.6, -3.83) |
| Stroke | -18.1 (-24.7, -12.9) | -31.3 (-41.9, -22.2) | -41.1 (-56.0, -28.7) | -33.8 (-45.9, -23.8) | -29.6 (-39.7, -21.1) | -36.6 (-172, -25.5) |
| Atrial fibrillation & flutter  **Cancer** | -142 (-201, -93.9) | -121 (-170, -80.3) | -119 (-168, -78.2) | -117 (-165, -77.7) | -112 (-158, -74.3) | -121 (-172, -79.5) |
| Colon & rectum Cancer  Esophageal  **Respiratory** | -2.10 (-2.89, -1.05)  -0.05 (-0.07, -0.04) | -3.56 (-5.14, -1,98)  -5.93 (-8.31, -3.96) | -3.14 (-4.68, -1.60)  -6.35 (-8.89, -4.21) | -3.44 (-5.16, -1.86)  -5.70 (-7.90, -3.85) | -3.58 (-5.30, -1.94)  -6.94 (-9.78, -4.55) | -1.71 (-2.52, -0.91)  < 0.01 (0.01, 0.01) |
| Asthma  **Musculo-skeletal** | -380 (-579, -205) | -363 (-558, -193) | -373 (-569, -200) | -329 (-500, -178) | -352 (-543, -184) | -458 (-713, -236) |
| Low back pain | -533 (-1044, -10.8) | -542 (-1096, -17.0) | -651 (-1270, -19.4) | -544 (-1045, -30.0) | -502 (-1045, 50.7) | -484 (-1033, 64.3) |
| Hip osteoarthritis  Knee osteoarthritis | 1.05 (1.05, 1.31)  5.51 (4.20, 6.56) | 0.79 (0.79, -1.19)  3.96 (3.16, 4.75) | 1.20 (0.94, 1.47)  5.34 (4.21, 6.55) | 1.03 (0.82, 1.24)  4.74 (3.71, 5.77) | 0.82 (0.67, 0.97)  3.88 (2.99, 4.70) | 0.50 (0.40, 0.61)  2.42 (1.82, 3.03) |
| **Females** | | | | | | |
| **Metabolic** |  |  |  |  |  |  |
| Type II diabetes | -1622 (-2049, -1235) | -1915 (-2400, -1474) | -1405 (-1778, -1059) | -1314 (-1657, -1000) | -1555 (-1952, -1199) | -1700 (-2174, -1245) |
| **Cardiovascular disease** |  |  |  |  |  |  |
| Ischaemic heart disease | -97.6 (-122, -75.7) | -77.0 (-95.8, -60.2) | -82.0 (-103, -63.3) | -80.6 (-101, -62.5) | -67.4 (-83.8, -52.7) | -61.5 (-77.3, -47.5) |
| Stroke | -37.3 (-50.3, -26.5) | -29.7 (-40.3, -21.1) | -39.1 (-53.9, -27.0) | -32.6 (-44.5, -22.7) | -29.8 (-40.1, -21.5) | -29.5 (-40.6, -20.3) |
| Hypertensive heart disease | -10.6 (-17.2, -4.50) | -11.7 (-18.0, -5.08) | -9.13 (-14.7, -3.31) | -10.5 (-16.6, -4.34) | -12.1 (-19.0, -5.54) | -8.53 (-14.4, -2.91) |
| Atrial fibrillation & flutter  **Cancer** | -64.0 (-90.5, -42.3) | -60.6 (-85.2, -40.3) | -54.9 (-77.7, -36.2) | -53.4 (-75.3, -35.3) | -62.4 (-88.1 -41.4) | -47.1 (-66.9, -31.0) |
| Colon & rectum Cancer  Esophageal  Breast Cancer  **Respiratory** | -2.12 (-2.91, -1.06)  <0.01 (0.01, 0.01)  -12.7 (-16.9, -8.73) | -1.95 (-2.74, -1.17)  -1.17 (-1.95, -0.78)  -12.9 (-17.2, -8.99) | -1.87(-2.73, -1.08)  <0.01 (0.01, 0.01)  -12.9 (-17.2, -8.99) | -1.93 (-2.89, -1.10)  -1.03 (-1.38, -0.69)  -13.0 (-17.4, -9.09) | -2.27 (-3.34, -1.28)  <0.01 (0.01, 0.01)  -13.1 (-17.5, -9.17) | -1.31 (-1.97, -0.75)  <0.01 (0.01, 0.01)  -12.3 (-16.4, -8.53) |
| Asthma  **Musculo-skeletal** | -781 (-1191, -420) | -635 (-965, -340) | -703 (-1069, -378) | -633 (-958, -343) | -615 (-945, -324) | -856 (-1329, -443) |
| Low back pain | -641 (-1272, 1.59) | -614 (-1216, -0.39) | -639 (-1248, -12.2) | -628 (-1210, -27.0) | -619 (-1258, 31.1) | -620 (-1295, 53.0) |
| Hip osteoarthritis  Knee osteoarthritis | 0.26 (0.26, 0.53  2.38 (1.85, 2.91) | 0.39 (0.39, 0.39)  1.95 (1.17, 2.35) | 0.36 (0.29, 0.43)  2.16 (1.65, 2.66) | 0.34 (0.28, 0.41)  1.93 (1.45, 2.34) | 0.28 (0.21, 0.36)  1.78 (1.35, 2.20) | 0.19 (0.09, 0.19)  0.94 (0.75, 1.22) |

Table S4: Change in incident cases of disease per 100,000 adult population (2018 to 2040), in specified local authorities as a result of the intervention, assuming 100% stringency
